# The Neurologist Lipman Halpern—Author of the *Oath of the Hebrew Physician*

**DOI:** 10.5041/RMMJ.10075

**Published:** 2012-04-30

**Authors:** Moshe Feinsod

**Affiliations:** Faculty of Medicine, Technion—Israel Institute of Technology, Haifa, Israel

**Keywords:** Hebrew, medical oath, neurology

## Abstract

Lipman Halpern was born in 1902 into a family of Grand Rabbis who lived in Bialystok from the mid-nineteenth century. Inspired by his son’s decision to study medicine, Halpern’s father authored a comprehensive and innovative book on medicine according to Rabbinic Law. After completing his initial medical studies in Königsberg, Halpern went on to specialize in neuropsychiatry in Berlin and then in Zurich.

In 1934, Halpern immigrated to Eretz-Israel (then Palestine), where he founded and expanded the Department of Neurology at the Hadassah University Hospital in Jerusalem. Under his guidance, the department became a leader in clinical neurology, clinical and basic neurological research, and teaching. For the graduation of the first class of the Faculty of Medicine of the Hebrew University of Jerusalem in 1952, he authored the “Oath of the Hebrew Physician,” which went on to become the official oath for all new physicians graduating from Israeli faculties of medicine.

Halpern authored many clinical and research articles in English, German, French, and Hebrew. His studies on the relationship between the vestibular, cerebellar, and visual systems resulted in the description of the phenomenon of “monocular disequilibrium” and the “sensorimotor induction syndrome,” also known as “Halpern’s syndrome.” In 1953 he became the first Israel Prize laureate in Medicine. Halpern died in 1968 while serving his second term as Dean of the Faculty of Medicine at Hebrew University.

## THE FAMILY HERITAGE

The Jewish population of the city of Bialystok grew considerably after the middle of the nineteenth century, and an 1897 census recorded that 63% of the city’s 66,000 citizens were Jewish. This growing community invited Rabbi Refael Yom-Tov Lipman Halpern, already famous for his scholarship and morals, to serve as their Chief Rabbi.

**Figure 1:**
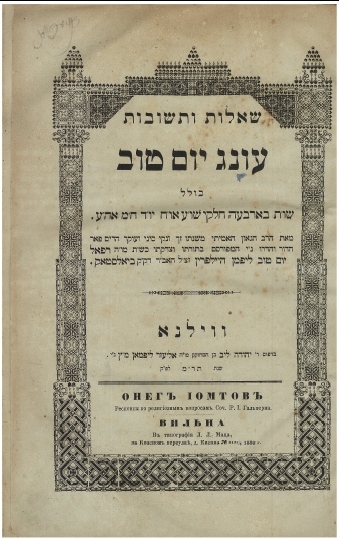
**Frontispiece of Rabbi Refael Yom-Tov Lipman Halpern book of Responsa *She'elot U-Teshuvot Oneg Yom Tov*, 1880.**

During his tenure (1859–1879) Rabbi Halpern authored a voluminous book of Responsa (*She'elot U-Teshuvot*) clarifying Jewish Law, which went on to become highly renowned throughout the rabbinical world (Figure 1). In 1961, over eighty years after its initial publication, the book was re-issued, attesting to its enduring relevance and value.

Rabbi Halpern’s son, Naphtali-Hertz, succeeded him in the position of Chief Rabbi, and his fame spread among Jews as well as Gentiles, to the extent that the bells of Bialystok’s churches tolled during his funeral. Naphtali-Hertz’s son, Rabbi Shlomo (Solomon) Halpern chaired the Rabbinical Court of Bialystok. He was quite disappointed when his two sons decided not to carry on the familial rabbinical line, but to pursue secular education at distant universities.

The eldest son, Lipman (named after his great-grandfather) went to study medicine in Königsberg, and the younger son, Israel, immigrated to Eretz-Israel (Palestine), studied history, and became Professor and Chair of the Department of History of the Jews in Poland, at the Hebrew University in Jerusalem.

To reconcile with the chosen path of his eldest son, in 1923 Rabbi Shlomo authored a treatise on Medicine and Jewish Law. The “Book of the Physicians” (*SeferHa Rofim*),^1^ written in classical Hebrew, is a comprehensive and highly original examination of contemporary medical studies, practices, attitudes, and ethics as viewed by Jewish Law (*Halacha*). The book emphasizes that devotion to the patient’s health and well-being overrides other directives and that the physician should be committed to continued learning and impeccable behavior. The handwritten manuscript was found posthumously among Lipman Halpern’s documents and was published in 1981 in *Assia*, a journal devoted to medicine and Jewish Law.^1^

Rabbi Shlomo continued to serve his congregation in Bialystok until late in June 1941. On that “Red Friday” the Germans gathered the city’s Jews—Rabbi Shlomo, their leader, among them—into the huge wooden synagogue and set it afire. More than 2,000 Jews perished in the blazing building.

## THE NEUROLOGIST LIPMAN HALPERN

Born in 1902, Lipman Halpern received an Orthodox Jewish education in Bialystok. He managed, however, to study secular subjects concomitantly at a state gymnasium. At the age of 21, Halpern left his home city to study medicine. Because of the notorious anti-Jewish quota (*numerus clausus*) practiced in Poland to curtail the number of Jewish university students, young Halpern enrolled in the medical faculty in Königsberg (now Kaliningrad), where a more liberal atmosphere prevailed.

After obtaining his medical degree in 1928, Halpern worked in the neuropsychiatric department and the physiological institute of that city. His main research interests and publications at the time addressed the electrophysiology of muscles and peripheral nerves, and the effect of drugs on the tremor of Parkinson’s disease.^2^ One of the drugs he tested was an alkaloid derivative, harmin (an MAO inhibitor), that had been suggested as a treatment for post-encephalitic Parkinson’s disease, but was alleged to have adverse psychiatric side-effects.^3^ Consistent with his high personal and ethical standards, Halpern tested the drug on himself, experienced its severe effects, and reported them in a scientific paper.^4^

In 1930, the famous neuropsychiatrist Kurt Goldstein (1873–1965), known for his studies on the effects of brain injuries in WWI survivors, and the originator of the *Gestalt* concept, moved to the *Moabit* Hospital in Berlin. After the famous *Charité*, the *Moabit* was the most important hospital in that city and was a center of Jewish physicians. Halpern joined Goldstein there and became interested in cerebral localization. In 1933, Goldstein was imprisoned by the Nazis and then expelled from Germany; at the same time Halpern fled to Zurich and worked for a year in the brain research institute there.

In 1934 he immigrated to Eretz-Israel and settled in Jerusalem. The following year, he married Adelhide (Adina) Gittelman, a musician and violin builder whom he knew from Königsberg and Berlin. Despite severe economic hardships, Halpern plunged into his professional work. He established a neuropsychiatric society and led its first scientific congress, then went on to initiate the first epidemiological study of psychiatric disorders among Jews and Arabs, in order to create a much-needed plan for the city’s hospitals and clinics.

After numerous struggles, in 1938 Halpern succeeded in establishing a neurological out-patient clinic at the Hadassah Hospital in Jerusalem, which also functioned as a teaching hospital for the growing Hebrew University. Within three years, an academic neurological department was established in the hospital, with Halpern serving as its first director, and with a curriculum for specialization in neuropsychiatry. Since his arrival to Eretz-Israel (Palestine) Halpern conducted research on frontal lobe injuries causing oculomotor disturbances,^5^ classification of epilepsy,^6^ and disturbances of the sense of position in various brain lesions.^7^ He also showed that the first language polyglots recover after aphasia is not necessarily the first that was learned (usually the mother tongue) but was often the language with the most profound emotional impact.^8^

Despite the privations caused by WWII, the small Jewish population, and the imminent danger of German forces advancing from Greece and Egypt, the Hebrew University and its hospital continued to establish departments and laboratories of the highest academic standards, with the vision of creating a medical center that would serve the entire Middle East. The hospital records show that in the early 1940s many Arab patients arrived from the neighboring countries. Halpern recognized the need for a neurosurgical department, convinced the administration, and helped found it in 1943.

During the War of Independence and the siege of Jerusalem, Halpern and the young neurosurgeon Aron Beller continued to make scientific observations on patients with head injuries. Despite all the hardships, the Faculty of Medicine marked its first graduating class in 1952. For the occasion, Halpern was asked to compose a new oath for physicians, in Hebrew, that would combine ancient Jewish culture and heritage with the spirit and vision of a modern Faculty of Medicine. Halpern’s oath took the form of ten directives and was written in beautiful Biblical Hebrew, inspired in style and spirit by his forefathers, the rabbinical dynasty of Bialystok. Through to this day, all graduates of medical faculties in Israel take this oath.

## THE OATH OF THE HEBREW PHYSICIAN

Novices of Medicine!

You stand this day before your masters in the ways of medicine and its statutes. That you should enter into covenant with medicine, to fulfill its laws with uprightness, and with all your might and mind that there may be established a generation of physicians worthy to do, and faithfully dedicated to succor the sick.

And this is the covenant which I make with you this day saying:

- You are charged day and night to stand by the sick in their distress at any time and at any hour.
- You shall watch verily over the life of man from his mother’s womb and his welfare shall always be your chief concern.
- And you will help the sick, base or honorable, stranger or alien or citizen, because he is sick.
- And you shall seek to fathom the soul of the sick, to restore their spirits with perspicacity and love of man.
- Do not hasten to bring forth judgment, and weigh your advice on a wise balance, tried in the crucible of wisdom forged by experience.
- Be loyal to those who put their trust in you. Reveal not his secret and go not as a taleteller.
- And make wise your heart to the health and welfare of the public and bring healing to alleviate the distress of the people.
- Give honor and esteem to your teachers who have striven to lead in the paths of medicine.
- Increase wisdom and weaken not, for wisdom is your life and from it shall flow the outcomes of life.
- Heed the dignity of your friends for in honoring them you will be honored.

The words of this covenant are most unto your mind and your heart to follow them with *your heart and with your whole soul* and you will answer—Amen.

**Amen thus we shall do.**

May your endeavors glorify the heritage of medicine in Israel.

## CONTINUED ACCOMPLISHMENTS: 1953–1968

In 1953, Halpern became the first recipient of the prestigious Israel Prize in Medicine for the discovery and elucidation of the “sensorimotor induction syndrome,” which came to be known as “Halpern’s syndrome.” This syndrome was described in detail by Halpern in a book that was published in Paris two years earlier.^9^ The book contained a multitude of observations and experiments Halpern conducted, beginning in the 1930s, and carried out amidst the struggle to build the Department of Neurology, his clinical work, and the upheavals of WWII and the War of Independence. Halpern’s syndrome defined the previously unrecognized influence of various sensory modalities on equilibrium, perception of the vertical and motor performance, as well as subjective sensations induced by colors in the presence of frontal lobe, vestibular, and cerebellar affections. Halpern’s observations were met at first with skepticism but were verified abroad and are even cited in our times.^10^–^14^

True to the teachings of his mentor, Kurt Goldstein, Halpern regarded neurology and psychiatry as one inseparable entity. In this spirit, in 1949, he became the medical director of the *Ezrat Nashim* psychiatric hospital in Jerusalem. There he introduced contemporary treatments such as electroshock therapy and lobotomy; the latter he abandoned out of dissatisfaction with the relatively lax indications that prevailed in the US at that time. He was deeply disturbed by the eventual separation of the neurological and psychiatric associations.^15^

**Figure 2:**
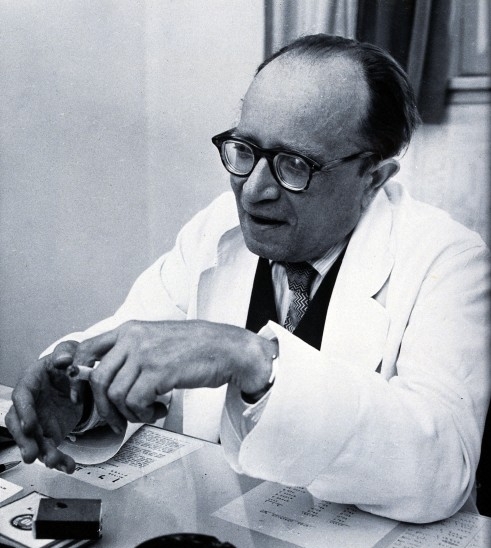
**Lipman Halpern (1902–1968).**

Under his leadership, the Department of Neurology at Hadassah University Hospital flourished, and new avenues of research were opened. An EEG and electrophysiology institute was established, as well as a laboratory of experimental neuroendocrinology and a center for neuroepidemiological research. The first major project of this last-mentioned center was a cross-country survey of multiple sclerosis. Halpern (Figure 2) reasoned that Israel, a country into which immigrants arrived from all over the world, could serve as a “laboratory” to study the influence of latitude and climate on the occurrence of MS on patients of diverse origins.^16^,^17^

Halpern was a cherished physician and teacher. He treated every patient, whether a top politician or the humblest individual, with the same warmth and diagnostic insight. He was a master of clinical teaching and was adored by his students as well as his staff. Halpern’s intimate acquaintance with Jewish Law and tradition, together with the wisdom of his forefathers and his excellent clinical standing, made him one of the best mediators between the Orthodox Jewish establishment and modern medicine. His contribution was crucial during the early years of the State of Israel.

Halpern’s achievements earned him recognition in the international neurology community; in 1953 he was elected to the Presidential Board of the International Congress of Clinical Neurology and, in 1957, to the Presidential Board of the First International Congress of the Neurological Sciences.

In 1963, Halpern published an international collection of essays, with contributions by the leading neurologists and neuropsychologists of that time, dealing with the localization and dynamics of the neurological “high functions.” The book continues to serve as a reference for issues such as referred pain, phantom pain, anosognosia, prosopagnosia, and sensorimotor induction syndrome.^18^ The Soviet Union forbade its scientists to contribute to this volume because of Halpern’s insistence that the book be published in Jerusalem.

As Dean, Halpern strove to strengthen the Faculty of Medicine, protect its position as the leading basic and applied research center, obtain financial support, and strengthen the contacts with its university hospital. His attitude, integrity, and warm personality were a source of confidence for faculty members, who elected him unanimously for a second term. Even as Dean, he continued his research on the higher cerebral functions.^19^ Halpern died of repeated heart attacks while in office, in 1968.

Halpern was succeeded as head of the Department of Neurology by his pupil, Professor Shaul Feldman, who later served as Dean of the Faculty of Medicine as well. This tradition of clinical and scientific excellence combined with public service was carried on by Feldman’s pupil, Professor Oded Abramsky, who also filled these two positions.

Halpern is survived by his daughter Rachel Halpern-Feinsod, MD.
